# Re-description of the type specimens of *Corinnommahamulatum* (Song & Zhu, 1992) stat. rest. (Araneae, Corinnidae) from Hubei, China

**DOI:** 10.3897/BDJ.13.e145705

**Published:** 2025-03-28

**Authors:** Xinxin Li, Chang Chu, Ye-Jie Lin

**Affiliations:** 1 University of Chinese Academy of Sciences, Beijing 100049, China University of Chinese Academy of Sciences Beijing 100049 China; 2 Key Laboratory of Zoological Systematics and Evolution, Institute of Zoology, Chinese Academy of Sciences, Beijing 100101, China Key Laboratory of Zoological Systematics and Evolution, Institute of Zoology, Chinese Academy of Sciences Beijing 100101 China; 3 Imperial College London, London, SW7 2AZ, United Kingdom Imperial College London London, SW7 2AZ United Kingdom

**Keywords:** Asia, diagnosis, morphology, spider, taxonomy

## Abstract

**Background:**

*Corinnomma* Karsch, 1880 comprises 14 species, distributed in Asia, Africa and America. Amongst Asian species, *Castianeirahamulata* Song & Zhu, 1992 was considered as a junior synonym of *Co.severum* (Thorell, 1877) by Deeleman-Reinhold (2001).

**New information:**

The species *Corinnommahamulatum* (Song & Zhu, 1992) **stat. rest.** is resurrected from its synonymy with *C.severum* (Thorell, 1877). Photos and morphological re-description of the type specimens are presented. The results of species delimitation using the Poisson Tree Process also support the resurrection of this species.

## Introduction

Family Corinnidae Karsch, 1880 comprises 76 genera and 876 known species on a global scale, encompassing two subfamilies: Castianeirinae Reiskind, 1969 and Corinninae Karsch,1880 (Reiskind 1969, World Spider Catalog 2025). The Castianeirinae includes 42 genera, which are typically mimics, usually of ants and occasionally of wasps (Silva-Junior and Bonaldo 2024).

*Corinnomma* Karsch, 1880, with the type species *Co.severum* (Thorell, 1877), belongs to the Castianeirinae and includes 14 species distributed across Asia, Africa and America (World Spider Catalog 2025). Amongst them, three species have been recorded from China: *Co.severum* (Thorell, 1877) (♂♀), *Co.simplex* L. Zhang, Jin & F. Zhang, 2022 (♂♀) and *Co.spirale* L. Zhang, Jin & F. Zhang, 2022 (♂♀) (World Spider Catalog 2025). This genus can be distinguished by the gradually sloping anterior abdomen, the dorsal scute being fused to the ventral scute and in the cylindrical palpal tibia without ventral concavity (Raven 2015). The species *Co.hamulatum* (=*Castianeirahamulata* Song & Zhu, 1992) is currently considered as a junior synonym of *Co.severum* (Deeleman-Reinhold 2001).

*Co.hamulatum* was originally described, based on two male and one female specimens from Hubei Province, China (Song and Zhu 1992). The original description was presented in Chinese and included inadequate figures (Song and Zhu 1992). Deeleman-Reinhold (2001) synonymised it with *Co.severum* without examining its type specimens. To resolve these taxonomic ambiguities, we re-studied the type specimens of *Co.hamulatum* to to verify its taxonomic validity.

## Materials and methods

All specimens were preserved in 80% ethanol. The female genitalia were cleared in trypsin enzyme solution to dissolve non-chitinous tissues. Specimens were examined under a LEICA M205C stereomicroscope. Photomicrographs were taken with an Olympus C7070 zoom digital camera (7.1 megapixels). Species distribution maps were generated using ArcView GIS 3.2 software (ESRI 2002).

All measurements are in millimetres and were obtained with an Olympus SZX16 stereomicroscope with a Zongyuan CCD industrial camera. Measurements follow Zhang et al. (2022). All measurements of body lengths do not include the chelicerae. Eye sizes are measured as the maximum diameter from either the dorsal or frontal view. Leg measurements are given as follows: total length (femur, patella, tibia, metatarsus, tarsus). The terminology used in the text and figures follows Haddad (2012) and Zhang et al. (2022). Abbreviations: ALE—anterior lateral eye; AME—anterior median eye; MOA—median ocular area; PLE—posterior lateral eye; PME—posterior median eye. Spination: do—dorsal; pl—prolateral; plv—prolateral ventral; rl—retrolateral; rlv—retrolateral ventral.

The material examined for this study is deposited in the following collections: **CYJL**—collection of Ye-Jie Lin, Dalian, China; **HNU**—College of Life Sciences, Hunan Normal University, Changsha, China; **IZCAS**—Institute of Zoology, Chinese Academy of Sciences, Beijing, China; **MHBU**—Museum of Hebei University, Baoding, China; **TRU**—Tongren University, Tongren, China.

In phylogenetic analysis, we included *Castianeira* sp. and Nyssuscf.coloripes as outgroups (Table 1). DNA was extracted from 2–4 legs using a TIANamp Genomic DNA Kit (TIANGEN Inc., Beijing, China) following the manufacturer’s protocol. Gene fragment were amplified in 25 μl reactions. Primers and PCR conditions for each locus followed Zhang et al. (2022). We used the Poisson Tree Process (PTP) for species delimitation (Zhang et al. 2013). The software was run on the RAxML COI gene tree (Suppl. material 1) for 2 × 10^6^ generations. Convergence was assessed in the output file.

## Taxon treatments

### Corinnomma hamulatum

(Song & Zhu, 1992)

CA8A9292-69A4-5767-AEFA-CDE6D11A214E

Castianeira hamulata Song & Zhu, 1992: 107, figs. 1–4 (♂♀); Song and Li (1997): 420, fig. 25A–D (♂♀); Song et al. (1999): 429, figs. 254O–P and 255E–F (♂♀).
Corinnomma severum (Thorell, 1877) - Deeleman-Reinhold (2001): 318, figs. 464–471 [♂♀, misidentification, considered as a senior synonym of *Castianeirahamulatum* and *Corinnommatiranglupa* (Barrion & Litsinger, 1995)]; Wang et al. (2012): 38, figs. 1A and 2A–I (♂♀, misidentification).
Castianeira severum Yin et al. (2012): 1131, fig. 601a–f (♂♀, misidentification).

#### Materials

- **Type status:** Holotype. **Occurrence:** catalogNumber: IZCAS-Ar9033; recordedBy: Anonymous; individualCount: 1; sex: female; lifeStage: adult; previousIdentifications: *Castianeirahamulata*; occurrenceID: 85711125-386B-5991-891C-0586F9ECD9E4; **Taxon:** scientificName: *Corinnommahamulatum*; **Location:** country: China; stateProvince: Hubei; county: Xuan'en; verbatimCoordinates: 109.7269°E, 29.7986°N; **Identification:** identifiedBy: Yejie Lin; dateIdentified: 2024; **Event:** year: 1989; month: 5; day: 25; **Record Level:** institutionCode: IZCAS
- **Type status:** Allotype. **Occurrence:** catalogNumber: IZCAS-Ar9034; recordedBy: Anonymous; individualCount: 1; sex: male; lifeStage: adult; previousIdentifications: *Castianeirahamulata*; occurrenceID: 377A8C84-90F4-5311-85FF-EA556EEA2A2E; **Taxon:** scientificName: *Corinnommahamulatum*; **Location:** country: China; stateProvince: Hubei; county: Xuan'en; verbatimCoordinates: 109.7269°E, 29.7986°N; **Identification:** identifiedBy: Yejie Lin; dateIdentified: 2024; **Event:** year: 1989; month: 5; day: 25; **Record Level:** institutionCode: IZCAS
- **Type status:** Paratype. **Occurrence:** catalogNumber: IZCAS-Ar9035; recordedBy: Anonymous; individualCount: 1; sex: male; lifeStage: adult; previousIdentifications: *Castianeirahamulata*; occurrenceID: 2DB91067-6DD2-5371-BAB6-D4521DE4A9CB; **Taxon:** scientificName: *Corinnommahamulatum*; **Location:** country: China; stateProvince: Hubei; county: Xianfeng; municipality: Maheba Village; verbatimElevation: 394 m; verbatimCoordinates: 109.2500°E, 29.7627°N; **Identification:** identifiedBy: Yejie Lin; dateIdentified: 2024; **Event:** year: 1989; month: 6; day: 4; **Record Level:** institutionCode: IZCAS
- **Type status:** Other material. **Occurrence:** recordedBy: Zhaoyi Li, Hui Wang, and Yang Chen; individualCount: 1; sex: male; lifeStage: adult; previousIdentifications: *Corinnomma* sp.; occurrenceID: 529E7D31-6697-5669-BECC-FD059F5F05C0; **Taxon:** scientificName: *Corinnommahamulatum*; **Location:** country: China; stateProvince: Hunan; county: Sangzhi; municipality: Xijie Forest Farm; verbatimCoordinates: 110.1826°E, 29.3945°N; **Identification:** identifiedBy: Yejie Lin; dateIdentified: 2024; **Event:** year: 2018; month: 11; day: 9; **Record Level:** institutionCode: MHBU
- **Type status:** Other material. **Occurrence:** recordedBy: Xiaoqi Mi, Hua Wang, and Mingyong Liao leg.; individualCount: 4; sex: 3 males, 1 female; lifeStage: adult; previousIdentifications: *Corinnommaseverum*; occurrenceID: A94A2EDF-43B2-50B0-A156-BF4F8B0AD634; **Taxon:** scientificName: *Corinnommahamulatum*; **Location:** country: China; stateProvince: Guizhou; county: Jiangkou; municipality: Fanjingshan National Nature Reserve; **Identification:** identifiedBy: Yejie Lin; dateIdentified: 2024; **Event:** year: 2011; month: 5; day: 11; **Record Level:** institutionCode: TRU
- **Type status:** Other material. **Occurrence:** recordedBy: Yongjing Zhang; individualCount: 1; sex: female; lifeStage: adult; previousIdentifications: *Castianeiraseverum*; occurrenceID: 266884E4-3390-570F-BFC2-102F46B26284; **Taxon:** scientificName: *Corinnommahamulatum*; **Location:** country: China; stateProvince: Hunan; county: Tongdao; municipality: Mujiao Township; **Identification:** identifiedBy: Yejie Lin; dateIdentified: 2024; **Event:** year: 1996; month: 6; day: 4; **Record Level:** institutionCode: HNU
- **Type status:** Other material. **Occurrence:** recordedBy: Liansuo Gong; individualCount: 1; sex: male; lifeStage: adult; previousIdentifications: *Castianeiraseverum*; occurrenceID: BC8E2DA1-3CA1-5878-BAD9-00ED8E367229; **Taxon:** scientificName: *Corinnommahamulatum*; **Location:** country: China; stateProvince: Hunan; county: Jiangyong; **Identification:** identifiedBy: Yejie Lin; dateIdentified: 2024; **Event:** year: 1991; month: 10; day: 13; **Record Level:** institutionCode: HNU
- **Type status:** Other material. **Occurrence:** recordedBy: Qingtian We; individualCount: 5; sex: 1 male, 4 females; lifeStage: adult; previousIdentifications: *Corinnomma* sp.; occurrenceID: AA1EEE86-99BD-5BFF-A60D-DACAB9B0BD46; **Taxon:** scientificName: *Corinnommahamulatum*; **Location:** country: China; stateProvince: Chongqing; county: Beibei; municipality: Jinyun Mountain; **Identification:** identifiedBy: Yejie Lin; dateIdentified: 2024; **Event:** year: 2021; month: 9; day: 23; **Record Level:** institutionCode: CYJL

#### Description

**Male (allotype)**. Total length 6.99 (7.05 with clypeus); carapace 3.42 long, 2.07 wide, opisthosoma 3.57 long, 2.14 wide. Eye sizes and interdistances: AME 0.18, ALE 0.11, PME 0.12, PLE 0.13; AME–AME 0.17, AME–ALE 0.05, PME–PME 0.26, PME–PLE 0.14, AME–PME 0.19, ALE–PLE 0.10. MOA 0.44 long, front width 0.45, back width 0.47. Clypeus height 0.15. Chelicerae with three promarginal (middle largest, distal smallest) and two retromarginal teeth (same size). Leg measurements: I 8.39 (2.26, 0.83, 2.07, 1.97, 1.26), II 7.67 (2.10, 0.78, 1.86, 1.77, 1.16), III 7.10 (1.94, 0.66, 1.76, 1.77, 0.97), IV 9.93 (2.68, 0.89, 2.24, 2.96, 1.16). Spination: femur I do 3 pl 1, II do 3, III do 3 pl 2 rl 2, IV do 3 pl 2 rl 2; patella III do 1; tibia I do 1 plv 3 rlv 3, II do1 plv 3 rlv 2, III plv 2 rlv 2 pl 1 rl 1, IV plv 2 rlv 2 pl 1 rl 1; metatarsi I–II plv 2 rlv 2, III plv 3 rlv 3 pl 2 rl 2, IV plv 3 rlv 3 pl 3 rl 3.

Colouration (Fig. 4A and B). Carapace medially reddish-brown, with dark-brown margins and covered with dense short white hairs. Eye region black. Fovea longitudinal, black. Radial furrow indistinct. Chelicerae reddish-brown. Endites reddish-brown. Labium broader than long, reddish-brown. Sternum reddish-brown, with truncated anterior margin and pointed posterior end. Coxae of legs I–II yellowish-brown, that of legs III–IV dark-brown, femora dark-brown, remaining segments reddish-brown, all covered with dense short white hairs. Opisthosoma grey-brown, pear-shaped, scutum strongly sclerotised, dorsal scutum covering almost 9/10 of opisthosoma, with yellow and white short hairs, ventral scutum covering almost 1/2 of opisthosoma, with white short hairs in the anterior region. Spinnerets reddish-brown.

**Figure 1.**
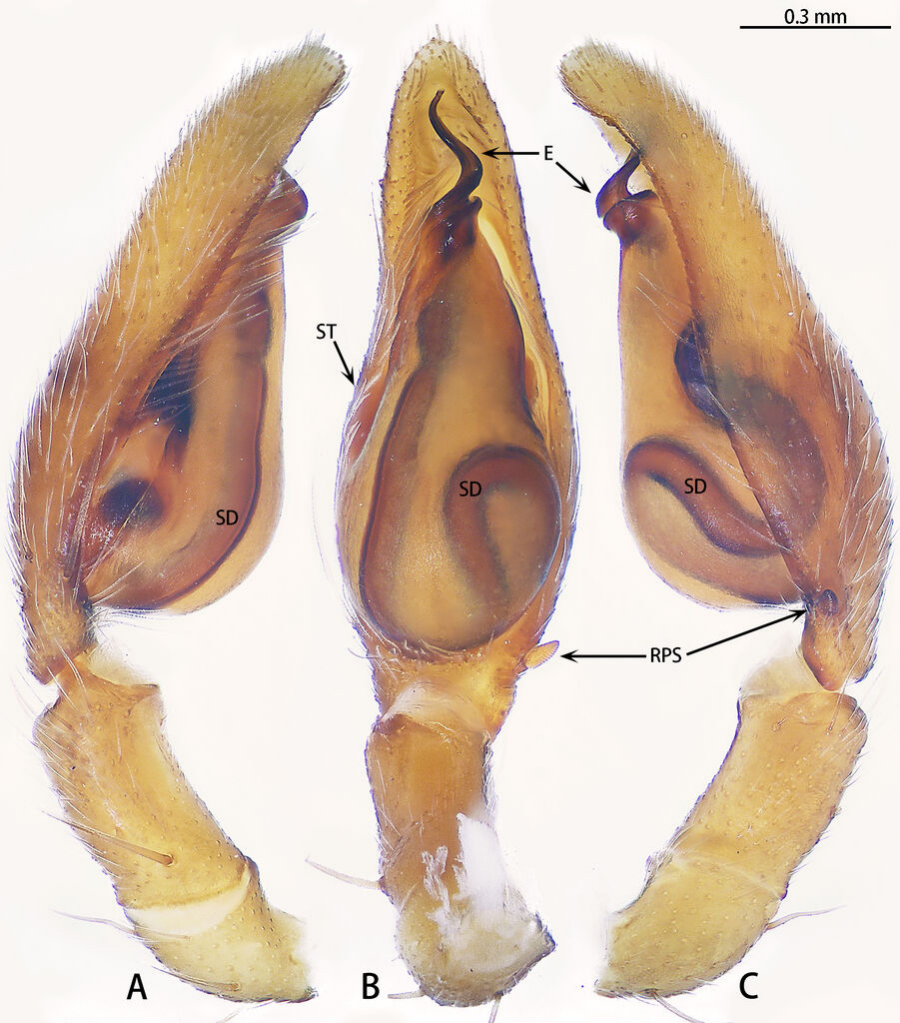
*Corinnommahamulatum* **stat. rest.**, allotype male. **A** Distal segments of palp, prolateral view; **B** Same, ventral view; **C** Same, retrolateral view. Abbreviations: E—embolus; SD—sperm duct; ST—subtegulum; RPS—Retrobasal paracymbial spine.

**Figure 2.**
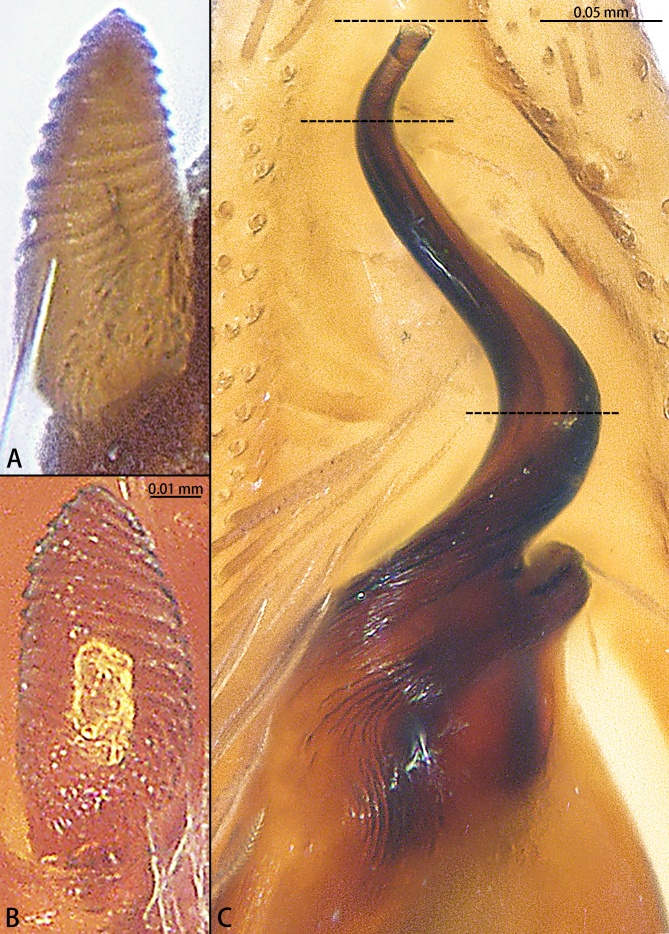
*Corinnommahamulatum* **stat. rest.**, allotype male. **A** Retrobasal paracymbial spine, lateral view; **B** Same, dorsal view; **C** Embolus, ventral view. Stipple lines indicate the demarcation between the basal and distal coils of the embolus.

Palp (Figs 1, 2). Femur with two spines dorsally, five times longer than wide. Patella slightly curved, 1.5 times longer than wide, with three spines dorsally. Tibia two times longer than wide, with a spine prolaterally, at one third of tibia. Cymbium almost three times longer than wide, with distinct subulate retrobasal paracymbial spine, without ridge. Subtegulum covered by tegulum, not obvious. Tegulum pear-shaped. Sperm duct with three curves, a constriction present near the embolus. Embolus S-shaped, helically twisted, with two bends in ventral view, almost 1/4 of the bulb.

**Female (holotype)**. Total length 8.26 (8.28 with clypeus); carapace 3.81 long, 2.46 wide, opisthosoma 4.45 long, 3.33 wide. Eye sizes and interdistances: AME 0.19, ALE 0.12, PME 0.12, PLE 0.12; AME–AME 0.21, AME–ALE 0.03, PME–PME 0.33, PME–PLE 0.18, AME–PME 0.19, ALE–PLE 0.12. MOA 0.43 long, front width 0.52, back width 0.58. Clypeus height 0.17. Chelicerae with three promarginal (middle largest, distal smallest) and two retromarginal teeth (same size). Leg measurements: I 8.97 (2.42, 0.91, 2.28, 2.07, 1.29), II 8.61 (2.42, 0.90, 2.06, 1.94, 1.29), III 7.68 (2.10, 0.85, 1.76, 1.87, 1.10), IV 11.15 (3.06, 1.02, 2.55, 3.17, 1.35). Spination: femur I do 4, II do 3 pl 1, III do 3 pl 1 rl 1, IV do 1 pl 1 rl 1; patella I do 1, III do 2; tibia I do 1 plv 2 rlv 2, II plv 2 rlv 3, III do 1 pl 2 rl 2, IV do 1 plv 3 rlv 3 pl 2 rl 2; metatarsi I–II plv 2 rlv 2, III plv 3 rlv 3 pl 2 rl 3, IV do 1 plv 3 rlv 3 pl 3 rl 3.

Colouration (Fig. 4C and D). As in male.

**Figure 3.**
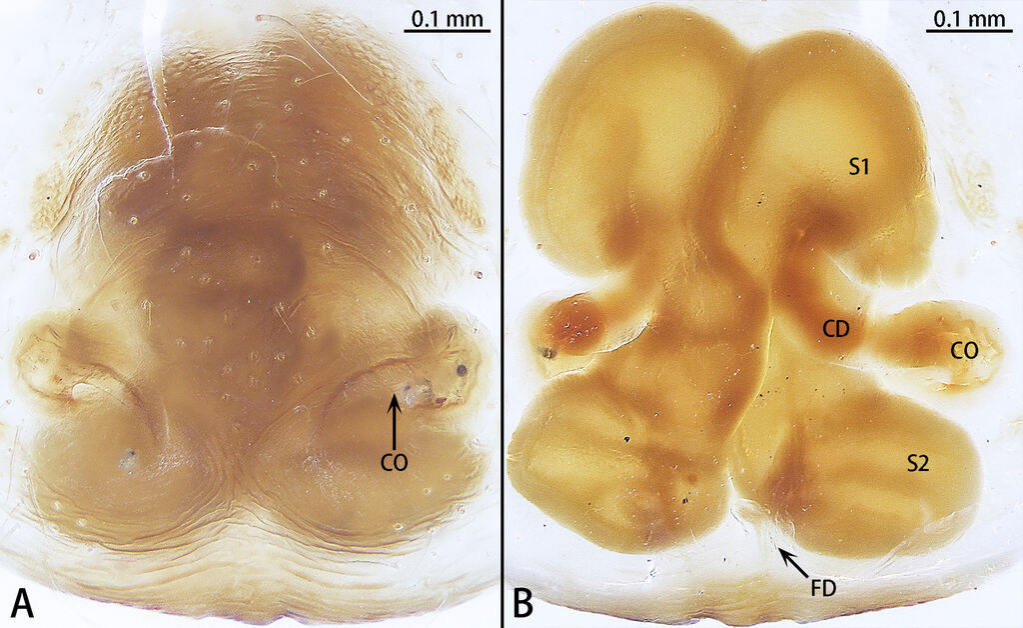
*Corinnommahamulatum* **stat. rest.**, holotype female. **A** Epigyne, ventral view; **B** Vulva, dorsal view. Abbreviations: CD—copulatory duct; CO—copulatory opening; FD—fertilisation duct; S1—spermatheca I; S2— spermatheca II.

Epigyne (Fig. 3A and B). Epigynal plate rectangular. Copulatory openings at middle of epigynal plate, with 6-shaped rims. Copulatory ducts C-shaped, attached to spermathecae I. Spermathecae I curved, contiguous. Spermathecae II sac-like, with straight, wrinkled and contiguous proximal and diverging globular distal parts. Fertilisation duct semi-circular, lying on Spermathecae II.

#### Diagnosis

*Corinnommahamulatum* **stat. rest.** resembles *C.severum* and *C.spirale* Zhang, Jin & Zhang, 2022 in having transverse stripes on the abdomen; male embolic tip curved and the length ratio of the basal coils to the distal coils is approximately 1:3; female with curved copulatory ducts.

However, the male can be distinguished by the retrobasal paracymbial spine conical, ridge absent (Fig. 2A and B) [vs. tongue-shaped, ridge present in *C.severum* (see Zhang et al. (2022): figs. 9A and B)], the length ratio of the embolus to the bulb is approximately 1:0.2 ventrally (Fig. 1B) [vs. 1:0.1 in *C.severum* [see Zhang et al. (2022): fig. 4B)], the distal fold almost 120° curved (Fig. 2C) (vs. 90° curved in *C.spirale* [see Zhang et al. (2022): fig. 10E)] and basal fold almost 90° curved ventrally (Fig. 2C) [vs. 110° curved in *C.severum* (see Zhang et al. (2022): fig. 4B)]. The females can be further distinguished by the length-to-width ratio of the copulatory opening being 1:0.4 (Fig. 3A) [vs. 1.0.6 in *C.severum* (see Zhang et al. (2022): fig. 4D)] and the C-shaped copulatory ducts (Fig. 3B) (vs. S-shaped in *C.spirale* (see Zhang et al. (2022): fig. 8D)].

#### Distribution

China (Hubei, Hunan, Chongqing, Guizhou) (Fig. 5).

#### Biology

Hunting during the day, mimicking ants. (Fig. 6).

#### Taxon discussion

COI-based phylogenetic analysis revealed two primary clades: one comprising *Corinnommaseverum* and the other encompassing three species (*C.simplex* Zhang, Jin & Zhang, 2022, *C.spirale* Zhang, Jin & Zhang, 2022 and *C.hamulatum* **stat. rest.**) (Suppl. material 1). Poisson Tree Processes (PTP) delimitation using 38 COI sequences identified six species (Table 1), excluding two outgroups (KY017624.1, KY017615.1). The delimited species include *C.simplex* (YNM112, YNM115), *C.spirale* (YNM106, YNM190, YNM111), *C.severum* (YNM116, YNM110, YNM113, YNM114, YNM162, YNM163, YNM164, YNM165, YNM167, YNM170, YNM175, YNM178, YNM180, YNM181, YNM182, YNM192, YNM193) and *C.hamulatum* **stat. rest.** (YNM169, YNM172, YNM173, YNM177, YNM183, YNM184, YNM185, YNM186, YNM187, YNM188, YNM189, YNM194, YNM195, YNM251). Statistical support for this delimitation was significant (Null-model score: 77.691; Best single coalescent rate score: 105.008; LRT p-value: < 0.001), showing partial congruence with previous findings in Zhang et al. (2022).

#### Notes

**Figure 4.**
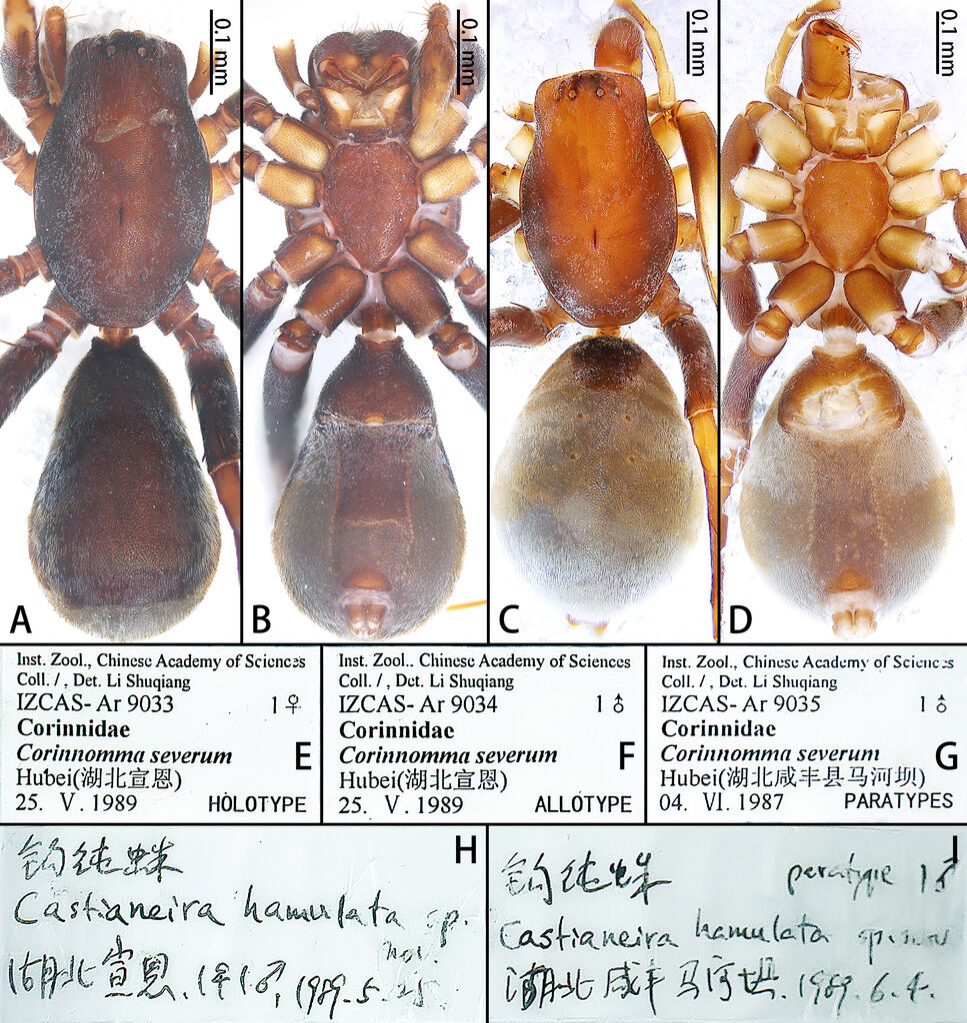
*Corinnommahamulatum* **stat. rest.**, habitus and labels, allotype male (**A**, **B**, **F, H**), holotype female (**C**, **D**, **E, H**) and paratype (**G, I**). **A** Allotype male, dorsal view; **B** Same, ventral view; **C** Holotype female, dorsal view; **D** Same, ventral view; **E–I** Original labels (**H**, **I** handwriting by Daxiang Song).

**Figure 5.**
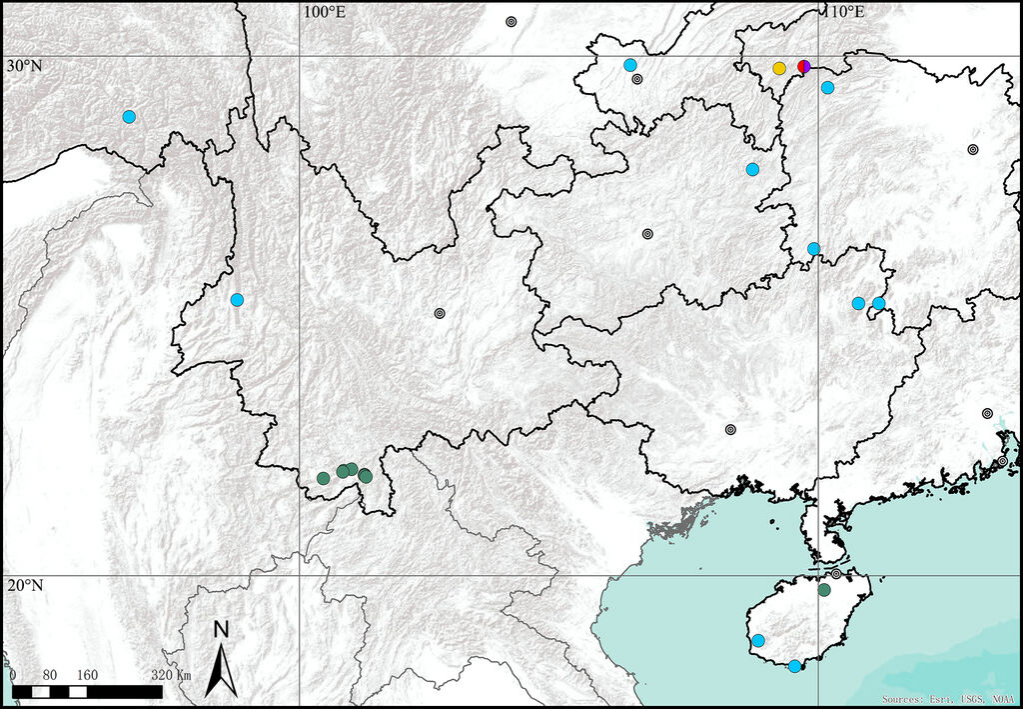
Distribution records of *Corinnomma* spp. *hamulatum* **stat. rest.** in China: *C.hamulatum* **stat. rest.**: **Red** holotype, **Purple** allotype, **Yellow** paratype, **Blue** other records; *C.severum*: **Green**.

**Figure 6.**
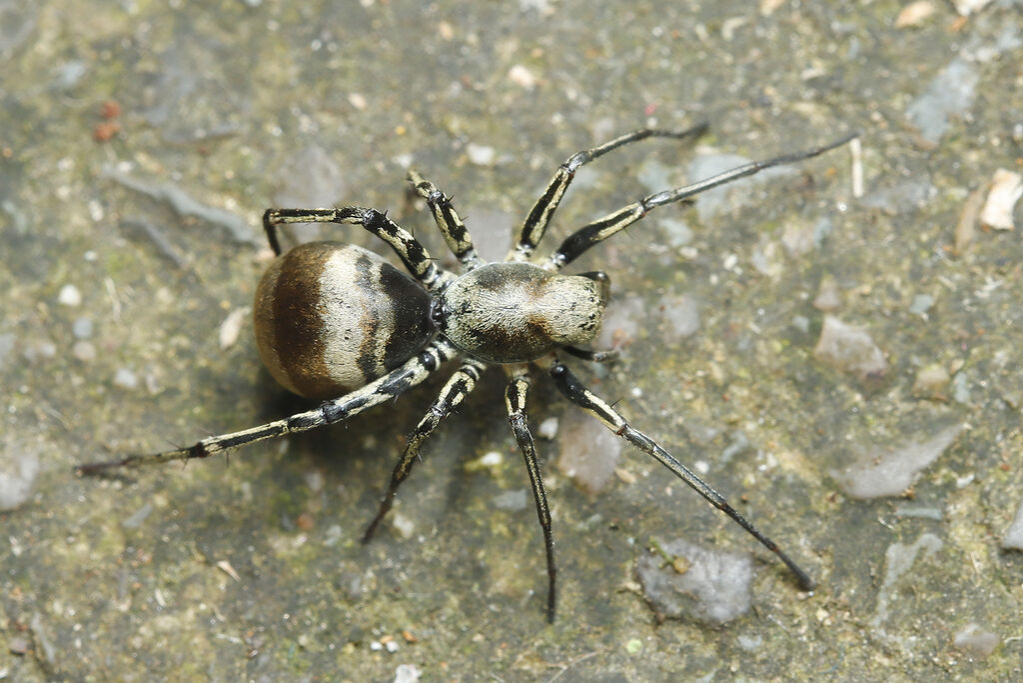
*Corinnommahamulatum* **stat. rest.**, female from Chongqing, in life. Photo by Qianle Lu.

As the paratype label does not match the handwritten label (Fig. 4G and I), the handwritten label takes precedence.

#### Comments

The recent re-examination of the type specimens of *Co.hamulatum* has revealed that it is different from *Co.severum* and should be recognised as a valid species. Consequently, the validity of *Co.hamulatum* **stat. rest.** is hereby restored.

## Supplementary Material

XML Treatment for Corinnomma hamulatum

4D7E59C7-C0BE-58AC-B89B-8C4A79E8DB18 10.3897/BDJ.13.e145705.suppl1 Supplementary material 1 RAxML COI gene tree, 38 specimens Data type phylogenetic tree Brief description Maximum Likelihood analysis was performed using RAxML v. 8.2.12 (Stamatakis 2014) with 1,000 ML Bootstrap replicates based on the GTRGAMMA model run by a rapid Bootstrap analysis. File: oo_1287302.pdf https://binary.pensoft.net/file/1287302 Xinxin Li

## Figures and Tables

**Table 1.** List of voucher information and GenBank accession numbers.

Taxon	Voucher	location	Genbank Accession Number (COI)
*Castianeira* sp.	MR527	Misiones, Argentina	KY017615.1
*Corinnommahamulatum*	YNM169	Yunnan, China	PV358000
*Corinnommahamulatum*	YNM172	Tibet, China	PV358002
*Corinnommahamulatum*	YNM173	Hainan, China	PV358003
*Corinnommahamulatum*	YNM177	Hainan, China	PV358005
*Corinnommahamulatum*	YNM183	Hainan, China	PV358010
*Corinnommahamulatum*	YNM184	Hainan, China	PV358011
*Corinnommahamulatum*	YNM185	Hainan, China	PV358012
*Corinnommahamulatum*	YNM186	Hainan, China	PV358013
*Corinnommahamulatum*	YNM187	Hainan, China	PV358014
*Corinnommahamulatum*	YNM188	Hainan, China	PV358015
*Corinnommahamulatum*	YNM189	Hainan, China	PV358016
*Corinnommahamulatum*	YNM194	Guangxi, China	PV358020
*Corinnommahamulatum*	YNM195	Hunan, China	PV358021
*Corinnommahamulatum*	YNM251	Hainan, China	PV358022
*Corinnommaseverum*	YNM116	Yunnan, China	ON054610
*Corinnommaseverum*	YNM110	Yunnan, China	ON054604
*Corinnommaseverum*	YNM113	Yunnan, China	ON054607
*Corinnommaseverum*	YNM114	Yunnan, China	ON054608
*Corinnommaseverum*	YNM162	Yunnan, China	PV357995
*Corinnommaseverum*	YNM163	Yunnan, China	PV357996
*Corinnommaseverum*	YNM164	Yunnan, China	PV357997
*Corinnommaseverum*	YNM165	Yunnan, China	PV357998
*Corinnommaseverum*	YNM167	Yunnan, China	PV357999
*Corinnommaseverum*	YNM170	Yunnan, China	PV358001
*Corinnommaseverum*	YNM175	Hainan, China	PV358004
*Corinnommaseverum*	YNM178	Yunnan, China	PV358006
*Corinnommaseverum*	YNM180	Yunnan, China	PV358007
*Corinnommaseverum*	YNM181	Yunnan, China	PV358008
*Corinnommaseverum*	YNM182	Yunnan, China	PV358009
*Corinnommaseverum*	YNM192	Yunnan, China	PV358018
*Corinnommaseverum*	YNM193	Yunnan, China	PV358019
*Corinnommasimplex*	YNM115	Yunnan, China	ON054609
*Corinnommasimplex*	YNM112	Yunnan, China	ON054606
*Corinnommaspirale*	YNM106	Yunnan, China	ON054603
*Corinnommaspirale*	YNM111	Yunnan, China	ON054605
*Corinnommaspirale*	YNM190	Yunnan, China	PV358017
Nyssuscf.coloripes	MR669	Tasmania, Australia	KY017624.1
