# Supplementary figures and images for: Re-description of the type specimens of Corinnommahamulatum (Song & Zhu, 1992) stat. rest. (Araneae, Corinnidae) from Hubei, China
Source: Biodivers Data J. 2025 Mar 28;13:e145705. doi: 10.3897/BDJ.13.e145705 (PMC11971639; doi:10.3897/BDJ.13.e145705)

KY017615.1

KY017624.1

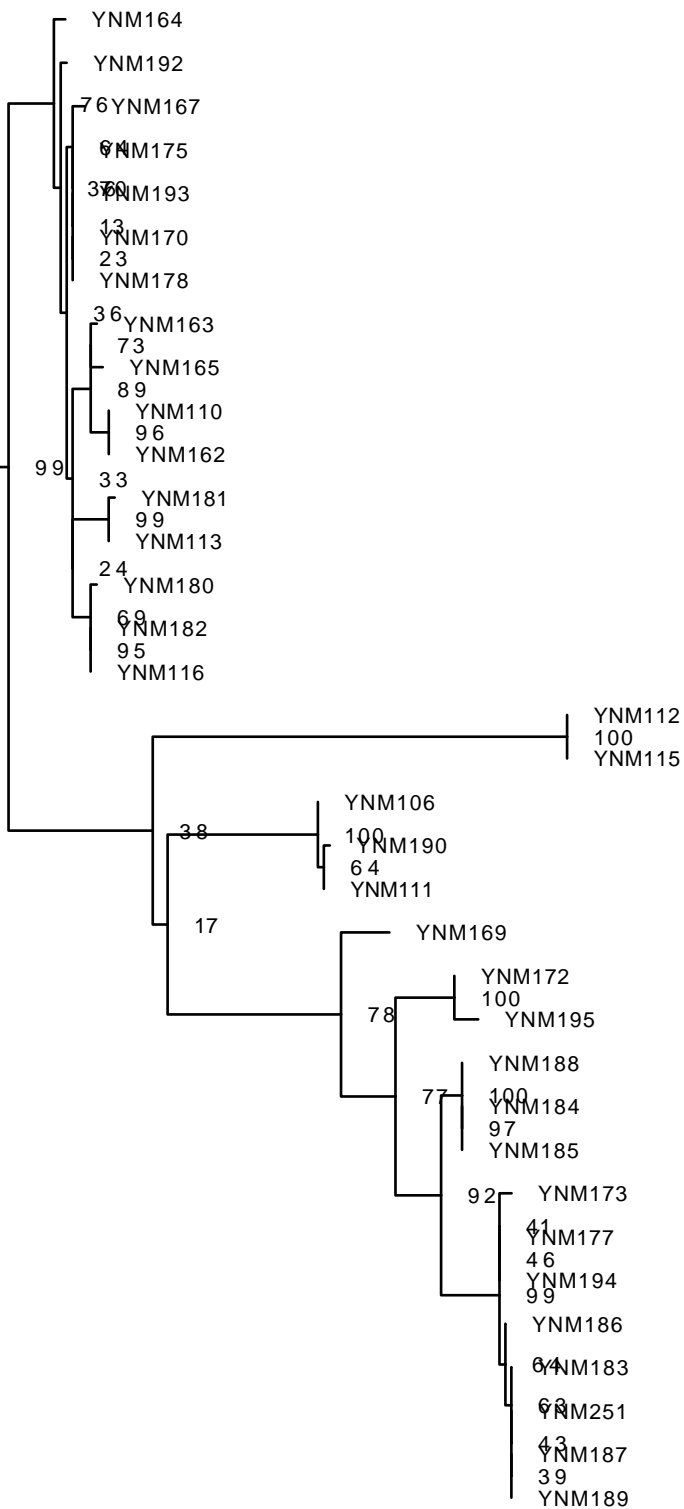

0.03

Supplement: Supplementary material 1 — RAxML COI gene tree, 38 specimens [file bdj-13-e145705-s001.pdf]
